# Relationships between Psychophysiological Responses to Cycling Exercise and Post-Exercise Self-Efficacy

**DOI:** 10.3389/fpsyg.2015.01775

**Published:** 2015-11-23

**Authors:** Eriko Matsuo, Shigeru Matsubara, Seigo Shiga, Kentaro Yamanaka

**Affiliations:** ^1^School of Pharmacy, Nihon UniversityFunabashi, Japan; ^2^Graduate School of Life Sciences, Showa Women’s UniversityTokyo, Japan; ^3^Institute of Women’s Health Sciences, Showa Women’s UniversityTokyo, Japan

**Keywords:** post-exercise self-efficacy, perceived exertion, heart rate variability, autonomic response, multiple regression analysis

## Abstract

Although self-efficacy (SE) is an important determinant of regular exercise, it is unclear how subjective and physiological states before, during, and after the exercise session affects post-exercise SE. The aim of this study was to clarify subjective and physiological factors affecting post-exercise SE assessed after a single exercise session at a physiologically equivalent level. Forty-three healthy volunteers (28 women, 15 men) completed an 82-min experimental session, comprising a 22-min pre-exercise rest, a 30-min steady-state cycling exercise at moderate intensity [40% of heart rate (HR) reserve], and a 30-min post-exercise rest. We measured physiological (HR) and subjective [Rating of Perceived Exertion (RPE), Feeling Scale (FS)] states during the experimental session. Autonomic states were assessed by power spectral analysis of heart rate variability (HRV) during pre- and post-exercise rest. Post-exercise SE, which was the participants’ confidence in their ability to perform the 30-min exercise that they had just performed, was assessed at 30-min post-exercise. A stepwise multiple regression analysis, with post-exercise SE as the dependent variable and physiological and subjective measures of the exercise as candidate explanatory variables, showed that post-exercise SE was negatively correlated with RPE and positively correlated with FS at the end of the 30-min exercise. In addition, post-exercise SE was negatively correlated with high-frequency power of the post-exercise HRV, an index of parasympathetic function. These results indicate that post-exercise SE is related not only to subjective responses to the exercise but also to autonomic response after the exercise.

## Introduction

The beneficial effects of exercise and physical activity on the development and maintenance of cardiorespiratory, musculoskeletal, and neuromotor fitness in most adults have been widely demonstrated (World Health Organization, 2010; Garber et al., 2011). Therefore, exercise quality and quantity recommendations have been included in the guidelines of the American College of Sports Medicine (ACSM) and the American Heart Association for healthy adults (Haskell et al., 2007). Nonetheless, physical inactivity remains a major public health problem in many industrialized countries (Kohl et al., 2012). Although regular exercise can clearly prevent many health problems, many find it difficult to adopt and adhere to prescribed exercise programs. This could be because, even when exercise intensity is defined at physiologically or self-selected equivalent levels, there are considerable inter-individual differences in physiological and subjective responses to exercise (Ekkekakis and Lind, 2006; Rose and Parfitt, 2007, 2010, 2012; Ekkekakis et al., 2009; DaSilva et al., 2011).

According to previous studies, exercise self-efficacy (SE) is an important determinant in the adoption and maintenance of a regular exercise regimen (McAuley and Blissmer, 2000). SE refers to the belief in one’s capability to successfully execute the actions necessary to satisfy specific situational demands (Bandura, 1997). McAuley et al. (2003) demonstrated that individuals with high exercise SE following a 6-months exercise program reported high activity levels at the 6- and 18-months follow-ups. Moreover, McAuley et al. (2007) demonstrated that many active individuals with high exercise SE at the 2-years follow-up reported still being active at the 5-years follow-up. Thus, high exercise SE was still associated with high physical activity levels for a considerable period afterward. Therefore, factors affecting exercise SE must be revealed to enable the promotion of prescribed exercise programs.

Bandura (1997) identifies four factors affecting SE: mastery experience, vicarious experience, social persuasion, and interpretations of physiological response. Previous studies suggest that exercise SE varies according to experiential and social factors: current exercise behavior or physical activity level (McAuley et al., 2003; Magnan et al., 2013), environmental support (McAuley et al., 2000, 2003), or pre-exercise SE (Pender et al., 2002). These results suggest that individuals who had a successful experience of and a better environment for exercise program performance strengthened their pre-exercise SE, which then promoted adherence to the exercise program easily. On the other hand, pre-exercise SE in individuals without successful exercise experience remains consistently low.

Previous studies demonstrated that affective responses to the exercise, which reflects interpretations of physiological response, also influenced exercise SE (Pender et al., 2002; McAuley et al., 2003; Focht et al., 2007; Kwan and Bryan, 2010; Magnan et al., 2013). For example, SE levels after the exercise increased (Katula et al., 1999; Pender et al., 2002) or decreased (Katula et al., 1999; Focht et al., 2007; Welch et al., 2010) compared with those before the exercise. This divergence was partly due to exercise intensity (Katula et al., 1999; Welch et al., 2010). These results indicated that exercise SE can be modified depending on individual subjective states during exercise, such as Rating of Perceived Exertion (RPE; Borg, 1982) and Feeling Scale (FS; Hardy and Rejeski, 1989), and the exercise experience can result in a successful experience with high exercise SE or an unsuccessful experience with low exercise SE. That is, an affective response to the exercise and the underlying physiological response might be one of the key factors in changing individual exercise SE.

It is well-known that the RPE provides a relatively good estimate of actual heart rate (HR) during exercise. In addition, it is generally believed that affective state correlates with autonomic nervous system activity (Ekman et al., 1983; Christie and Friedman, 2004; Kreibig, 2010). Spectral analysis of heart rate variability (HRV) is an established non-invasive tool that can be used to study the autonomic control of HR at rest (TaskForce, 1996; Perini and Veicsteinas, 2003; Sandercock and Brodie, 2006). For example, using the HRV indices, Sakuragi and Sugiyama (2006) indicated that regular walking exercise improved mood states and shifted autonomic balance to parasympathetic predominance. Weinstein et al. (2007) reported that reduced parasympathetic activity in the baseline period correlated with the increases in negative mood symptoms in the exercise-withdrawal group. From these results, we considered that autonomic activity might be related to subjective responses to exercise and post-exercise SE. However, it is unclear how physiological measures before, during, and after the exercise session relate to post-exercise SE, directly or indirectly. Therefore, as the first step for examining the detailed relationship between interpretations of physiological response to exercise and post-exercise SE, it is important to focus the subjective (RPE and FS) and/or physiological measures (HRV indices) against a steady-state exercise session at a physiologically equivalent intensity level.

The aim of the present study was to clarify the subjective and/or physiological (especially autonomic) factors affecting post-exercise SE assessed after a single exercise session at a physiologically equivalent level. We hypothesized that post-exercise SE is related to not only subjective but also physiological (especially autonomic) measures. For this purpose, we measured subjective and physiological responses to a 30-min steady-state cycling exercise performed at moderate intensities and recorded post-exercise SE 30 min after the exercise. In order to focus the effect of the subjective and/or physiological (especially autonomic) responses to the exercise on post-exercise SE, we asked no question about experiential and social factors beforehand. Furthermore, only post-exercise SE was assessed with the intention of minimizing the aftereffect of self-reported pre-exercise SE. Next, we attempted to identify significant factors affecting post-exercise SE among the measured subjective and physiological variables. However, it is unclear what physiological variables relate to post-exercise SE and how subjective variables interact with them. Therefore, we used a stepwise multiple regression analysis as an exploratory method. In this analysis, post-exercise SE was the dependent variable and physical characteristics and subjective and physiological responses to the exercise were candidate explanatory variables.

## Materials and Methods

### Participants

Forty-three healthy volunteers (28 women, 15 men, age range: 18–24 years-old) not taking any medication or undergoing treatment were sampled. They were recruited from undergraduate student populations at Showa Women’s University and Nihon University. All participants signed an informed consent document before participating in an experimental session and measured their body weight and height (InBody J10; Biospace, Co., Ltd., Seoul, Korea) to calculate their body mass index (BMI). Participants were asked to have a caffeine-free meal at least 2 h before visit to our laboratory. We confirmed by interview that they did not engage in regular exercise, competitive sports, or manual labor, and did not use bicycle ergometer frequently. After completing all experimental procedures, they received a monetary reward. This study was approved by the ethics committee of Showa Women’s University and School of Pharmacy, Nihon University.

### Post-exercise Self-efficacy

Our post-exercise SE scale was modified from McAuley’s Exercise Self-Efficacy Scale (McAuley et al., 1993), and comprised three items on participants’ confidence in their ability to perform the 30-min exercise that they had just performed. The items included (1) “I can perform the 30-min exercise three to five times per week at a 10% increased level of intensity from the level I just maintained,” (2) “I can perform the 30-min exercise three to five times per week at the same level of intensity as the level I just maintained,” and (3) “I can perform the 30-min exercise three to five times per week at a 10% reduced level of intensity from the level I just maintained.” Participants answered each item on an 11-point scale ranging from 0% (*not at all confident*) to 100% (*highly confident*). Before the experimental session, one of the authors (EM) explained to participants the method for assessing post-exercise SE. Participants were instructed to rate the SE scales based on their confidence about their execution of the exercise that they had just performed. The average of the responses to the three-item questionnaire was used in the data analysis. McAuley’s Exercise Self-Efficacy Scale demonstrated good internal consistency (α = 0.92; McAuley et al., 1993). Our measures also demonstrated good internal consistency (α = 0.92).

### Subjective Measures

Participants’ subjective states were assessed using the RPE (Borg, 1982) and the FS (Hardy and Rejeski, 1989). One of the authors (EM) orientated each participant regarding completion of the RPE and FS just before an experimental session. The 15-point RPE scale, ranging from 6 (*no exertion at all*) to 20 (*maximal exertion*), was used to estimate perceived whole-body exertion. The 11-point FS, which ranges from –5 (*very bad*) to +5 (*very good*), measured basic or core affective valence (pleasant – unpleasant).

### Experimental Session

**FIGURE 1:**
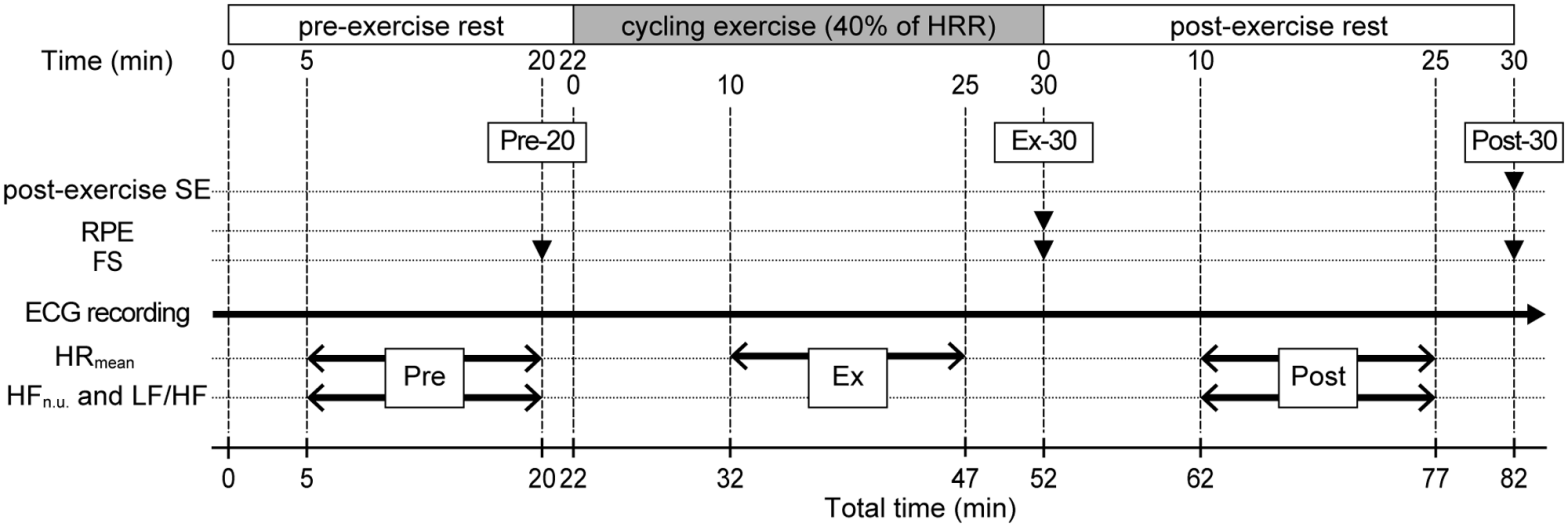
**Experimental protocol.** An 82-min experimental session comprised a 22-min pre-exercise rest (Pre), a 30-min exercise (Ex), and a 30-min post-exercise rest (Post). Filled triangles indicate the time points for recording of subjective measures represented on the left. Two-headed arrows indicate the time periods for steady-state HR data represented on the left. SE, self-efficacy; RPE, rating of perceived exertion; FS, Feeling Scale; %HRR, percentage of heart rate reserve.

Each participant conducted a single experimental session individually, and the experiment was performed under the control of two or more researchers. An experimental session lasted 82 min, comprising a 22-min pre-exercise rest (Pre), a 30-min exercise (Ex), and a 30-min post-exercise rest (Post; **Figure 1**). Participants breathed freely, and their HRs were continuously monitored throughout the session. Participants exercised on an electrically braked bicycle ergometer (Aerobike 900U-ex, Konami Sports & Life, Co., Ltd., Tokyo, Japan). Exercise intensity was set at 40% of HR reserve (HRR). Thus, the target HR during exercise for each participant was calculated as follows: target HR = resting HR + 0.4 × (maximal HR – resting HR). Maximal HR was calculated as 220 – age. Resting HR was the mean HR value during a 15-min Pre period.

The experimental session began after participants’ 10-min rest on a chair to familiarize themselves with the experimental setting. During the Pre period, participants sat in a chair next to a bicycle ergometer. Twenty minutes after the start of the Pre period (Pre-20), participants recorded their FS, then moved from the chair to the bicycle ergometer. At Pre-22, participants started a bicycle exercise. One of the authors (EM) gradually increased the bicycle’s workload during the first 5 min of the Ex period and adjusted it so the participants could exercise at their target HR levels. Afterward, while participants’ HR were monitored, the workload was adjusted every 5 min during the Ex period. This enabled the participants to perform steady-state exercise at their target HR levels (maximal adjustment range was ±10W in a single session). Thirty minutes after the start of the Ex period (Ex-30), participants completed the bicycle exercise and immediately recorded their RPE and FS on the ergometer. After that, they moved from the ergometer to the chair, sitting in the chair in a relaxed state during the Post period. Thirty minutes after the start of the Post period (Post-30), participants recorded their FS and experiential post-exercise SE. We recorded RPE and FS only at the end of each component of the session (only at Ex-30 for RPE, and at Pre-20, Ex-30, and Post-30 for FS) because it was easy to focus self-report measurements for the participants.

### Physiological Data Recording and Analysis

Throughout the experimental session, participants’ HRs were continuously monitored using a three-lead electrocardiogram (ECG; BSM-2401 ECG monitor, Nihon Kohden, Tokyo, Japan). The output signal from the ECG monitor, a train of rectangular impulses corresponding to the QRS spikes, was stored sequentially by a bio-amplifier recording device (Polymate II AP216, TEAC, Japan) and used for the subsequent off-line analysis (sampling rate = 1,000 Hz). The period between consecutive heartbeats (R–R intervals: RRI) was calculated using all of the output signal data collected during an 82-min experimental session. Next, a trained researcher (one of the authors, KY) searched the RRI data for outliers, which would likely have been caused by bodily movements during the exercise, and corrected these by either omitting (for extra beats) or inserting beats (for doubled or tripled beats). The mean percentage of abnormal beats during an 82-min experimental session was 0.23%; most of these were observed during the transition between the chair and the bicycle ergometer. From the corrected RRI data, steady-state RRI data (5–20 min in the Pre period, 10–25 min in the Ex period, and 10–25 min in the Post period) were extracted and converted to HR data (beats/min), and the average HR was calculated for each steady-state 15-min period (HR_mean_). Actual %HRR values in the Ex period were then calculated.

Next, the steady-state RRIs were interpolated and re-sampled at 10 Hz using a cubic spline function to obtain equally spaced samples. Then, power spectral analysis of the HRV data was performed using a fast Fourier transform algorithm. The data were categorized into high-frequency (HF; 0.15–0.40 Hz) and low-frequency (LF; 0.04–0.15 Hz) bands and calculated as integrals under the respective power spectral density functions (ms^2^). HF power in normalized units [HFn.u. = HF/(LF + HF) × 100] was calculated as an index of parasympathetic function, and the LF/HF ratio was calculated as an index of sympathovagal balance (TaskForce, 1996). These data analyses were performed using Matlab R2012b (MathWorks, Natick, MA, USA).

### Statistical Analysis

As a prerequisite for examining the relation between post-exercise SE and subjective and physiological measures, we needed to confirm whether the experiment was carried out as planned (participant characteristics and experimental settings) and how participants responded to the exercise subjectively and physiologically. Therefore, we first conducted two-sample *t*-tests on the participant characteristics and exercise parameters for women and men. Next, we examined temporal changes of subjective (FS) and physiological measures (HR_mean_, HF_n.u._, and LF/HF) as follows. On FS and HR_mean_, a repeated measures ANOVA was conducted with three time points (Pre-20, Ex-30, and Post-30) or periods (Pre, Ex, and Post) as the within-subjects factor. For the repeated measures ANOVA, we used Mauchly’s test to evaluate the sphericity assumption, and, when necessary, a Greenhouse–Geisser procedure was used to correct the degrees of freedom. If necessary, *post hoc* multiple comparisons were conducted by using paired *t*-tests with Bonferroni correction. On the HRV indices in Pre- and Post-exercise resting periods (HFn.u. and LF/HF during Pre and Post), paired *t*-tests were conducted. Finally, in order to identify some of the factors contributing to post-exercise SE, we conducted a stepwise multiple regression analysis. We chose post-exercise SE as the dependent variable and participants’ physical characteristics (BMI), physiological and subjective states during pre-exercise rest (HR_mean_, HF_n.u._, and LF/HF in Pre and FS at Pre-20), exercise (HR_mean_ in Ex and RPE and FS at Ex-30), and post-exercise rest (HR_mean_, HF_n.u._, and LF/HF in Post and FS at Post-30) as candidate explanatory variables. All statistical analyses were performed using SPSS Statistics 19.0 (IBM SPSS, Chicago, IL, USA). The data were presented as mean ± SD.

## Results

### Participant Characteristics and Exercise Settings

In **Table 1**, participant characteristics (age, height, weight, and BMI) and exercise settings (workloads of bicycle ergometer and %HRR) are shown for women (*n* = 28) and men (*n* = 15). Although there were significant sex differences in height (*t*[41] = 7.17, *p* < 0.01, *d* = 2.32) and weight (*t*[41] = 4.21, *p* < 0.01, *d* = 1.25), no significant differences were observed regarding BMI (*t*[41] = 0.84, *p* = 0.41, *d* = 0.26). Four participants were categorized as “underweight” (from 16.0 to 18.5) and five were “overweight” (from 25 to 30), while 34 participants were categorized as “healthy weight” (from 18.5 to 25). No participants were “severely underweight” (BMI < 16.0) or “obese” (BMI ≥ 30). Next, there was a significant sex difference in workloads of bicycle ergometer (*t*[41] = 5.50, *p* < 0.01, *d* = 1.58), while there was no significant difference in %HRR (*t*[41] = 0.85, *p* = 0.40, *d* = 0.40). These results provide strong evidence that most participants had normal physical characteristics, and both female and male participants exercised at relatively equivalent intensity as planned. Since there was no significant sex difference on age, BMI, and %HRR, we pooled all participants’ (women and men) data in the following analyses.

**Table 1:** Participant characteristics and exercise parameters.

	Female (*n* = 28)	Male (*n* = 15)
Age (year)	21.0 ± 1.2	20.7 ± 1.7
Height (cm)	158.9 ± 4.7	169.4 ± 4.4^∗^
Weight (kg)	53.2 ± 5.5	62.3 ± 8.7^∗^
BMI (kg/m^2^)	21.1 ± 2.0	21.7 ± 2.6
Workload (W)	70.4 ± 11.3	99.3 ± 23.3^∗^
%HRR	40.3 ± 2.0	39.3 ± 3.1

*Data presented as mean ± SD. BMI, bodymass index. ^∗^*p* < 0.05; Unpaired *t*-tests show significant differences between male and female participants*.

### Subjective and Autonomic Responses to Steady-state Cycling Exercise

Subjective and physiological responses to exercise are shown in **Table 2**. A repeated measures ANOVA on FS scores revealed no significant effect of time points (*F*[2,42] = 2.02, *p* = 0.13, $\eta_{p}^{2}$ = 0.05). This indicates that FS scores had relatively large individual differences and did not change in a uniform way for all participants. A repeated measures ANOVA on HR_mean_ revealed a significant effect of time periods (*F*[2,42] = 2960.5, *p* < 0.01, $\eta_{p}^{2}$ = 0.98), and *post hoc* multiple comparison revealed that there were significant differences in the HR_mean_ between Pre and Ex (*t*[42] = -63.3, *p* < 0.01, *d* = 10.33), Ex and Post (*t*[42] = 58.9, *p* < 0.01, *d* = 9.60), and Pre and Post periods (*t*[42] = -6.29, *p* < 0.01, *d* = 0.58). This indicates that HR changed similarly for all participants according to exercise session, and the HR_mean_ in Post did not completely return to the baseline level (the HR_mean_ in Pre). A paired *t*-test revealed that HF_n.u._ (index of parasympathetic function) was significantly larger in Post than in Pre (*t*[42] = 2.93, *p* < 0.01, *d* = 0.26), and the LF/HF ratio (index of sympathovagal balance) was significantly smaller in Post than in Pre (*t*[42] = -2.58, *p* < 0.05, *d* = 0.35). These results indicate that autonomic states were different before and after the exercise. The post-exercise SE values at Post-30 were around 70 with a substantial inter-individual difference (range: 20–100).

**Table 2:** Subjective and physiological responses to exercise and post-exercise SE.

		Pre-exercise rest	Exercise	Post-exercise rest
Subjective responses	RPE		13.0 ± 2.1	
	FS	1.28 ± 1.56	1.88 ± 1.53	1.49 ± 1.74
Physiological responses	HR_mean_ (bpm)	69.6 ± 6.5	121.4 ± 2.9	73.3 ± 6.5^#^
	HF_n.u._	0.40 ± 0.17		0.36 ± 0.16^∗^
	LF/HF	1.91 ± 1.11		2.43 ± 1.78^∗^
Post-exercise SE (%)				69.4 ± 23.4

*SE, Self-efficacy; RPE, Rating of Perceived Exertion; FS, Feeling Scale; HF_*n.u.*_, high-frequency power of heart-rate variability in normalized unit; LF, low-frequency power of heart-rate variability. Data are mean ± SD. #*p* < 0.05; Two-way factorial ANOVA shows a significant main effect of time period. ^∗^*p* < 0.05; Paired *t*-tests show significant differences between pre- and post-exercise rest*.

### Stepwise Multiple Regression Analysis

**FIGURE 2:**
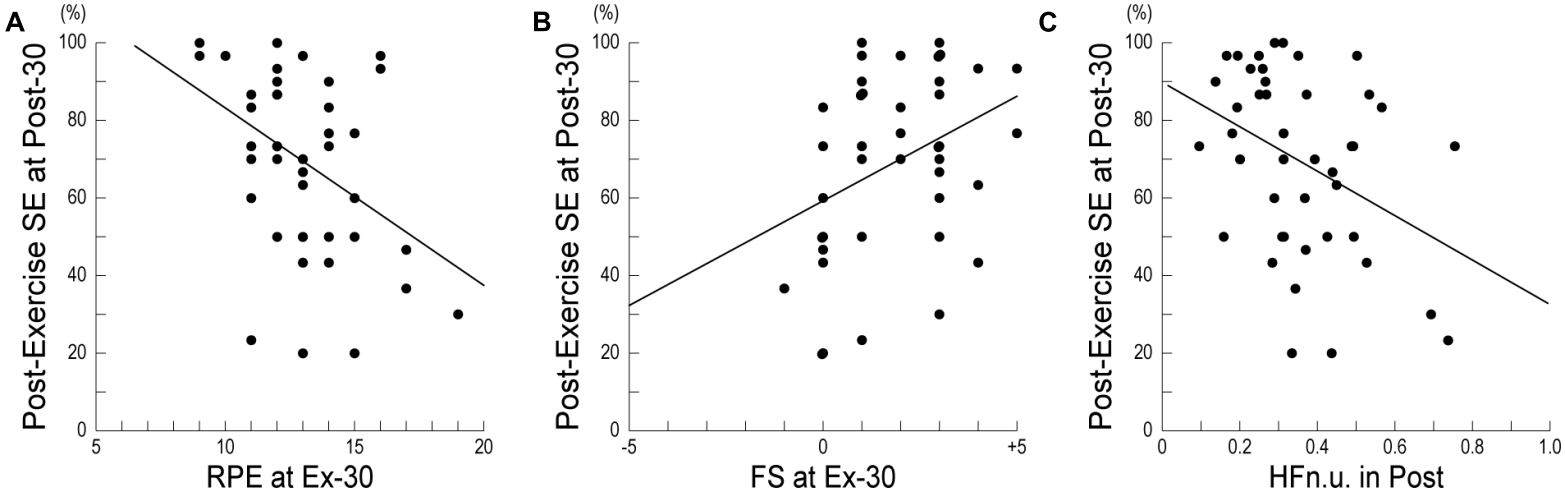
**Relationship between post-exercise self-efficacy and subjective or physiological measures. (A)** Relationship between post-exercise SE and RPE at Ex-30 (*r* = 0.167, *p* < 0.05). **(B)** Relationship between post-exercise SE and FS at Ex-30 (*r* = 0.125, *p* < 0.05). **(C)** Relationship between post-exercise SE and HF_n.u._ in Post (*r* = 0.145, *p* < 0.05). SE, Self-Efficacy; RPE, Rating of Perceived Exertion; FS, Feeling Scale; HF_n.u._, high-frequency power of heart-rate variability in normalized unit.

**Table 3** shows the results of a stepwise multiple regression analysis for post-exercise SE at Post-30. In the final model, the post-exercise SE at Post-30 was negatively correlated with RPE at Ex-30, positively correlated with FS at Ex-30, and negatively correlated with HF_n.u._ in Post. **Figure 2A** shows the relationships between post-exercise SE at Post-30 and RPE at Ex-30; participants with high RPE scores at Ex-30 assessed their post-exercise SE at Post-30 as low. **Figure 2B** shows the relationships between post-exercise SE at Post-30 and FS at Ex-30; participants with high FS scores at Ex-30 assessed their post-exercise SE at Post-30 as high. **Figure 2C** shows the relationships between post-exercise SE at Post-30 and HF_n.u._ in Post; participants with high HF_n.u._ (index of parasympathetic activity) in Post assessed their post-exercise SE at Post-30 as low. These results indicate that, when participants reported low RPE and high FS at the end of the exercise and revealed low parasympathetic activity during post-exercise resting period, they assessed post-exercise SE at Post-30 as high.

**Table 3:** Result of stepwise multiple regression analysis.

Step	Adjusted *R*^2^	*F*	Explanatory variable	*B*	*SE*	β	*t*
**Dependent variable: post-exercise SE at Post-30**							
Step 1	0.146	8.192	RPE at Ex-30	-4.569	1.596	-0.408	-2.861
Step 2	0.542	8.308	RPE at Ex-30 FS at Ex-30	-4.602 5.440	1.488 2.029	-0.411 0.356	-3.093 2.681
Step 3	0.347	8.445	RPE at Ex-30 FS at Ex-30 HFn.u. in Post	-3.846 5.626 -48.697	1.427 1.905 19.170	-0.343 0.369 -0.324	-2.694 2.954 -2.540

*SE, Self-efficacy; adjusted *R*^*2*^, coefficient of determination adjusted for degrees of freedom; *B*, partial regression coefficient; *SE*, standard error; β, standardized partial regression coefficient; RPE, Rating of Perceived Exertion; FS, Feeling Scale; HF_*n.u.*,_ high-frequency power in normalized units; Ex, exercise; Post, post-exercise rest*.

## Discussion

The aims of the present study were to clarify subjective and/or physiological factors affecting post-exercise SE. To achieve this purpose, we recorded subjective (RPE and FS) and physiological (HR_mean_ and HRV indices) responses to a 30-min steady-state cycling exercise at moderate intensity (40% of HRR) and post-exercise SE at 30 min post-exercise. Stepwise multiple regression results suggest that post-exercise SE at the end of the 30 min post-exercise period was mainly dependent on RPE and FS at the end of the exercise and on HF_n.u._ during post-exercise resting period. To our knowledge, this is the first study revealing the relationship between post-exercise SE and not only subjective but also autonomic measures.

We first conducted a careful check to ensure all participants exercised at the planned intensity levels (40% of HRR) and obtained strong evidence that all participants exercised as planned. Therefore, other variables examined in this study (RPE, FS, and HRV indices) were considered representative of participants’ subjective and physiological responses to a 30-min steady-state cycling exercise at the target moderate intensity. Despite such a strict setting of physiologically equivalent exercise intensity, FS scores at Ex-30 had a large inter-individual variability, which is similar to previous results (Rose and Parfitt, 2007, 2010; Haile et al., 2013). On the other hand, Ekkekakis and Lind (2006) and Ekkekakis et al. (2009) showed that the pattern of change in the affective responses differed among normal-weight, overweight, and obese individuals. In our study, however, only five participants were categorized as “overweight,” and no participants were “obese.” Therefore, it is not likely that BMI was a main factor in the inter-individual FS variability observed in this study. Moreover, post-exercise SE had a substantial inter-individual difference (range: 20–100). This also lends strong support to our hypothesis that post-exercise SE can be altered by many subjective and physiological factors.

This study’s “moderate” level was relatively low (range: 40–59% of HRR) in ACSM’s exercise intensity classification (Garber et al., 2011). Since we did not recruit participants based on their physical fitness levels, it is highly probable that inactive individuals were included. Previous study demonstrated that, when controlling for physiological values (oxygen uptake or HR) during exercise, individuals with high fitness levels perceived themselves to be under less exertion than did those with low fitness levels (Travlos and Marisi, 1996). Another study reported that, when controlling for subjective value (RPE), individuals with low fitness levels exhibited greater physiological indices during exercise than did those with high fitness levels (Kaufman et al., 2006). That is, we assumed that relatively low exercise intensity might be suitable for an investigation of subjective and/or physiological factors affecting experiential post-exercise SE among our participants with wide range of physical fitness levels. In fact, our participants’ RPE scores were relatively high in the above-mentioned ACSM classification (RPE 12–13).

With respect to the physiological measures used during pre- and post-exercise resting periods, HR_mean_ was significantly higher in the Post than in the Pre period. In addition, HF_n.u._ (index of parasympathetic function) was significantly larger in Post than in Pre and the LF/HF ratio (index of sympathovagal balance) were significantly smaller in Post than in Pre. These results indicate that, probably due to physiological aftereffects of the exercise, participants were sympathetic dominant during post-exercise resting periods after a 30-min steady-state cycling exercise at moderate intensity. This diversity in subjective (RPE and FS) and physiological (HR_mean_ and HRV indices) responses to the exercise might be one of the factors of a substantial inter-individual difference in post-exercise SE. Therefore, it is important to reveal factors affecting post-exercise SE using exploratory stepwise multiple regression analyses.

Stepwise multiple regression analysis showed that post-exercise SE at Post-30 was negatively correlated with RPE and positively correlated with FS, at Ex-30. This result indicates that participants with strong perceptions of exertion and bad feelings during exercise typically assessed their confidence in their ability to perform the 30-min exercise that they had just performed as low. This relationship between post-exercise SE and RPE at Ex-30 is similar to a previous finding that RPE scores during exercise partly explained the variance in post-exercise SE (Pender et al., 2002). The interesting aspect of this result is that an extracted affecting factor of post-exercise SE was not HR_mean_ during exercise, which directly reflects exercise intensity, but RPE and FS at the end of the exercise, which indirectly reflects relative exercise intensity through participants’ subjective states. That is, participants’ post-exercise SE is more strongly influenced by subjective states of physical stress, effort, and fatigue than by physiological cardiovascular states during exercise. Furthermore, post-exercise SE at Post-30 was negatively correlated with HF_n.u._ in Post. This significant relationship between post-exercise SE and HRV indices was critically interesting. When we consider that the HF_n.u._ is an index of parasympathetic function, this suggests that participants with high vagal activity (and low sympathetic activity) during the post-exercise resting period tended to assess their post-exercise SE as low. This result suggests that post-exercise SE might be related to the balance of autonomic HR control in the post-exercise resting period.

In this study, we focused one of Bandura’s four factors affecting SE: interpretations of physiological response. Therefore, we hypothesized that post-exercise SE is related to physiological and subjective measures to the exercise and focused on the relationships between interpretations of physiological response to the exercise and post-exercise SE. As a result, we demonstrated that post-exercise SE is related not only to subjective responses to exercise but also to autonomic response after the exercise. On the other hand, previous studies reported that other social and experiential factors affect post-exercise SE (McAuley et al., 2000, 2003; Magnan et al., 2013). In addition, pre-exercise SE affected subjective responses to the following exercise (Katula et al., 1999; Jerome et al., 2002; Focht et al., 2007; Magnan et al., 2013). Moreover, it is not clear whether the level of post-exercise SE remains stable over time, especially by the time of the next exercise participation. That is, it is important to examine how post-exercise SE, which is modified according to the exercise participation influences many factors affecting exercise SE afterward. As a next step, therefore, we will try to reveal interactive relationships between pre- and post-exercise SE and many other factors.

Several methodological limitations of this study should be discussed. First, our sample size was small. For multiple regression analysis of our experimental setting (effect size f^2^ [large] = 0.35, α = 0.05, power = 0.8, number of predictors [explanatory variables] = 12), sample size was *a priori* estimated to 61. On the other hand, statistical power from *post hoc* computing using our data (effect size f^2^ = 0.39) was 0.61. Based on the small sample size and its statistical power, we have to be careful in interpreting our regression analysis. In addition, the age range of our sample was narrow (18–24 years-old), and none of the participants were of an extreme physical size (BMI: 17.1–26.3). Moreover, we did not collect data on their daily physical activity levels. Further research should be conducted with a larger and more diverse sample, and participant characteristics should be examined closely. Second, the exercise intensity (%HRR) used in this study was based not on a ventilatory threshold but on HR. Since affective (Rose and Parfitt, 2007) and autonomic (Yamamoto et al., 1992) responses during exercise differ above and below one’s ventilatory threshold, participants’ post-exercise SE may have been affected by whether they surpassed their ventilatory threshold. Third, since applying traditional HRV analysis to exercise HR data remains controversial (Perini and Veicsteinas, 2003; Sandercock and Brodie, 2006), we assessed HRV indices only during pre- and post-exercise resting periods and did not address HRV indices during exercise. Some improved methods for HRV analysis during exercise have been proposed, such as coarse-graining spectral analysis (Yamamoto and Hughson, 1991), Poincaré plot (Tulppo et al., 1999), and time-frequency analysis using short-time Fourier transforms (Cottin et al., 2004; Pichon et al., 2004). Applying these analyses will provide us with information on autonomic HR control during exercise.

In summary, we demonstrated that SE regarding an exercise that participants had just performed is related to exercise-induced subjective and autonomic responses. In particular, one of the contributor to post-exercise SE was HF_n.u._ in the Post-resting period, which is considered an index of autonomic HR control. This is the first study to examine the relationship between post-exercise SE and HRV indices (which probably reflect autonomic control) after the exercise. Although further research is needed to confirm the methodological validity and reproducibility of these results, the present findings contribute to our understanding of the interactive relationship between post-exercise SE and physiological and subjective states before and during exercise.

## Conflict of Interest Statement

The authors declare that the research was conducted in the absence of any commercial or financial relationships that could be construed as a potential conflict of interest.

## References

[B1] Bandura A. (1997). *Self-Efficacy: The Exercise of Control.* New York, NY: Freeman.

[B2] Borg G. A. (1982). Psychophysical bases of perceived exertion. *Med. Sci. Sports Exerc.* 14 377–381. 10.1249/00005768-198205000-00012 7154893

[B3] Christie I. C. Friedman B. H. (2004). Autonomic specificity of discrete emotion and dimensions of affective space: a multivariate approach. *Int. J. Psychophysiol.* 51 143–153. 10.1016/j.ijpsycho.2003.08.002 14693364

[B4] Cottin F. Médigue C. Leprêtre P. M. Papelier Y. Koralsztein J. P. Billat V. (2004). Heart rate variability during exercise performed below and above ventilatory threshold. *Med. Sci. Sports Exerc.* 36 594–600. 10.1249/01.MSS.0000121982.14718.2A 15064586

[B5] DaSilva S. G. Guidetti L. Buzzachera C. F. Elsangedy H. M. Krinski K. De Campos W. (2011). Psychophysiological responses to self-paced treadmill and overground exercise. *Med. Sci. Sports Exerc.* 43 1114–1124. 10.1249/MSS.0b013e318205874c 21088625

[B6] Ekkekakis P. Lind E. (2006). Exercise does not feel the same when you are overweight: the impact of self-selected and imposed intensity on affect and exertion. *Int. J. Obes.* 30 652–660. 10.1038/sj.ijo.0803052 16130028

[B7] Ekkekakis P. Lind E. Vazou S. (2009). Affective responses to increasing levels of exercise intensity in normal-weight, overweight, and obese middle-aged women. *Obesity* 18 79–85. 10.1038/oby.2009.204 19556979

[B8] Ekman P. Levenson R. W. Friesen W. V. (1983). Autonomic nervous system activity distinguishes among emotions. *Science* 221 1208–1210. 10.1126/science.6612338 6612338

[B9] Focht B. C. Knapp D. J. Gavin T. P. Raedeke T. D. Hickner R. C. (2007). Affective and self-efficacy responses to acute aerobic exercise in sedentary older and younger adults. *J. Aging Phys. Act.* 15 123–138. 17556780 10.1123/japa.15.2.123

[B10] Garber C. E. Blissmer B. Deschenes M. R. Franklin B. A. Lamonte M. J. Lee I. M. (2011). American College of Sports Medicine position stand: quantity and quality of exercise for developing and maintaining cardiorespiratory, musculoskeletal, and neuromotor fitness in apparently healthy adults: guidance for prescribing exercise. *Med. Sci. Sports Exerc.* 43 1334–1359. 10.1249/MSS.0b013e318213fefb 21694556

[B11] Haile L. Goss F. L. Robertson R. J. Andreacci J. L. Gallagher M. Jr. Nagle E. F. (2013). Session perceived exertion and affective responses to self-selected and imposed cycle exercise of the same intensity in young men. *Eur. J. Appl. Physiol.* 113 1755–1765. 10.1007/s00421-013-2604-0 23412542

[B12] Hardy C. J. Rejeski W. J. (1989). Not what, but how one feels: the measurement of affect during exercise. *J. Sport Exerc. Psychol.* 11 304–317.

[B13] Haskell W. L. Lee I. M. Pate R. R. Powell K. E. Blair S. N. Franklin B. A. (2007). Physical activity and public health: updated recommendation for adults from the American College of Sports Medicine and the American Heart Association. *Med. Sci. Sports Exerc.* 39 1423–1434. 10.1249/mss.0b013e3180616b27 17762377

[B14] Jerome G. J. Marquez D. X. McAuley E. Canaklisova S. Snook E. Vickers M. (2002). Self-efficacy effects on feeling states in women. *Int. J. Behav. Med.* 9 139–154. 10.1207/S15327558IJBM0902_05 12174532

[B15] Katula J. A. Blissmer B. J. McAuley E. (1999). Exercise intensity and self-efficacy effects on anxiety reduction in healthy, older adults. *J. Behav. Med.* 22 233–247. 10.1023/A:1018768423349 10422616

[B16] Kaufman C. Berg K. Noble J. Thomas J. (2006). Ratings of perceived exertion of ACSM exercise guidelines in individuals varying in aerobic fitness. *Res. Q. Exerc. Sport* 77 122–130. 10.1080/02701367.2006.10599338 16646359

[B17] Kohl H. W. I. I. I. Craig C. L. Lambert E. V. Inoue S. Alkandari J. R. Leetongin G. (2012). The pandemic of physical inactivity: global action for public health. *Lancet* 380 294–305. 10.1016/S0140-6736(12)60898-8 22818941

[B18] Kreibig S. D. (2010). Autonomic nervous system activity in emotion: a review. *Biol. Psychol.* 84 394–421. 10.1016/j.biopsycho.2010.03.010 20371374

[B19] Kwan B. M. Bryan A. D. (2010). Affective response to exercise as a component of exercise motivation: attitudes, norms, self-efficacy, and temporal stability of intentions. *Psychol. Sport Exerc.* 11 71–79. 10.1016/j.psychsport.2009.05.010 20161385 PMC2782828

[B20] Magnan R. E. Kwan B. M. Bryan A. D. (2013). Effects of current physical activity on affective response to exercise: physical and social-cognitive mechanisms. *Psychol. Health* 28 418–433. 10.1080/08870446.2012.733704 23088712 PMC3593984

[B21] McAuley E. Blissmer B. (2000). Self-efficacy determinants and consequences of physical activity. *Exerc. Sport Sci. Rev.* 28 85–88. 10902091

[B22] McAuley E. Blissmer B. Katula J. Duncan T. E. (2000). Exercise environment, self-efficacy, and affective responses to acute exercise in older adults. *Psychol. Health* 15 341–355. 10.1080/08870440008401997

[B23] McAuley E. Jerome G. J. Elavsky S. Marquez D. X. Ramsey S. N. (2003). Predicting long-term maintenance of physical activity in older adults. *Prev. Med.* 37 110–118. 10.1016/S0091-7435(03)00089-6 12855210

[B24] McAuley E. Lox C. Duncan T. E. (1993). Long-term maintenance of exercise, self-efficacy, and physiological change in older adults. *J. Gerontol.* 48 218–224. 10.1093/geronj/48.4.P218 8315239

[B25] McAuley E. Morris K. S. Motl R. W. Hu L. Konopack J. F. Elavsky S. (2007). Long-term follow-up of physical activity behavior in older adults. *Health Psychol.* 26 375–380. 10.1037/0278-6133.26.3.375 17500625

[B26] Pender N. J. Bar-Or O. Wilk B. Mitchell S. (2002). Self-efficacy and perceived exertion of girls during exercise. *Nurs. Res.* 51 86–91. 10.1097/00006199-200203000-00004 11984378

[B27] Perini R. Veicsteinas A. (2003). Heart rate variability and autonomic activity at rest and during exercise in various physiological conditions. *Eur. J. Appl. Physiol.* 90 317–325. 10.1007/s00421-003-0953-9 13680241

[B28] Pichon A. P. de Bisschop C. Roulaud M. Denjean A. Papelier Y. (2004). Spectral analysis of heart rate variability during exercise in trained subjects. *Med. Sci. Sports Exerc.* 36 1702–1708. 10.1249/01.MSS.0000142403.93205.35 15595290

[B29] Rose E. A. Parfitt G. A. (2007). A quantitative analysis and qualitative explanation of the individual differences in affective responses to prescribed and self-selected exercise intensities. *J. Sport Exerc. Psychol.* 29 281–309. 17876968 10.1123/jsep.29.3.281

[B30] Rose E. A. Parfitt G. A. (2010). Pleasant for some and unpleasant for others: a protocol analysis of the cognitive factors that influence affective responses to exercise. *Int. J. Behav. Nutr. Phys. Act.* 7 15 10.1186/1479-5868-7-15 PMC2832617 20181111

[B31] Rose E. A. Parfitt G. A. (2012). Exercise experience influences affective and motivational outcomes of prescribed and self-selected intensity exercise. *Scand. J. Med. Sci. Sports* 22 265–277. 10.1111/j.1600-0838.2010.01161.x 20626702

[B32] Sakuragi S. Sugiyama Y. (2006). Effects of daily walking on subjective symptoms, mood and autonomic nervous function. *J. Physiol. Anthropol.* 25 281–289. 10.2114/jpa2.25.281 16891758

[B33] Sandercock G. R. H. Brodie D. A. (2006). The use of heart rate variability measures to assess autonomic control during exercise. *Scand. J. Med. Sci. Sports* 16 302–313. 10.1111/j.1600-0838.2006.00556.x 16774653

[B34] TaskForce (1996). Heart rate variability: standards of measurement, physiological interpretation and clinical use. Task Force of the European Society of Cardiology and the North American Society of Pacing and Electrophysiology. *Circulation* 93 1043–1065. 10.1161/01.CIR.93.5.1043 8598068

[B35] Travlos A. K. Marisi D. Q. (1996). Perceived exertion during physical exercise among individuals high and low in fitness. *Percept. Mot. Skills* 82 419–424. 10.2466/pms.1996.82.2.419 8724910

[B36] Tulppo M. P. Makikallio T. H. Laukkanen R. T. Huikuri H. V. (1999). Differences in autonomic modulation of heart rate during arm and leg exercise. *Clin. Physiol.* 19 294–299. 10.1046/j.1365-2281.1999.00180.x 10451789

[B37] Weinstein A. A. Deuster P. A. Kop W. J. (2007). Heart rate variability as a predictor of negative mood symptoms induced by exercise withdrawal. *Med. Sci. Sport Exerc.* 39 735–741. 10.1249/mss.0b013e31802f590c 17414813

[B38] Welch A. S. Hulley A. Beauchamp M. (2010). Affect and self-efficacy responses during moderate-intensity exercise among low-active women: the effect of cognitive appraisal. *J. Sport Exerc. Psychol.* 32 154–175. 20479476 10.1123/jsep.32.2.154

[B39] World Health Organization (2010). *Global Recommendations on Physical Activity for Health.* Available at: http://whqlibdoc.who.int/publications/2010/9789241599979_eng.pdf 26180873

[B40] Yamamoto Y. Hughson R. L. (1991). Coarse-graining spectral analysis: new method for studying heart rate variability. *J. Appl. Physiol.* 71 1143–1150. 1757311 10.1152/jappl.1991.71.3.1143

[B41] Yamamoto Y. Hughson R. L. Nakamura Y. (1992). Autonomic nervous system responses to exercise in relation to ventilatory threshold. *Chest* 101(5 Suppl.), 206S–210S. 10.1378/chest.101.5_Supplement.206S 1576836

